# Etymologia: *Neospora caninum*

**DOI:** 10.3201/eid2506.ET2506

**Published:** 2019-06

**Authors:** Ronnie Henry

**Keywords:** *Neospora caninum*, parasites, sporozoan parasite, coccidian, cattle, dogs

## *Neospora caninum* [ne-osʹpə-rə ca-ninʹum]

From the *neo*- (Latin, “new”) + *spora* (Greek, “seed”) and *canis* (Latin, “dog”), *Neospora caninum* (Figure) is a sporozoan parasite that was first described in 1984. It is a major pathogen of cattle and dogs but can also infect horses, goats, sheep, and deer. Antibodies to *N. caninum* have been found in humans, predominantly in those with HIV infection, although the role of this parasite in causing or exacerbating illness is unclear.

**Figure:**
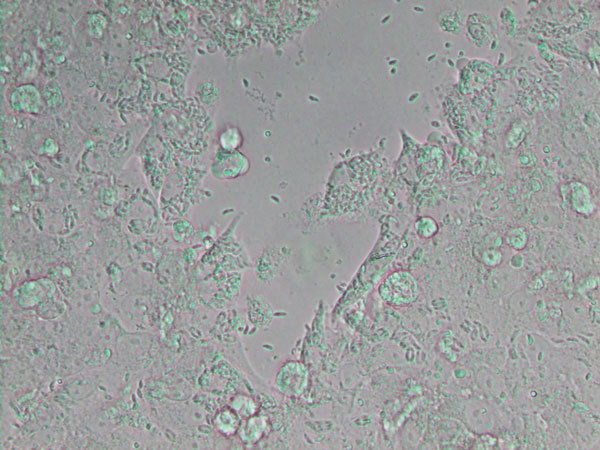
*Neospora caninum*, a coccidian parasite, which identified as a species in 1988. It is a major cause of spontaneous abortion in infected livestock. Image from WIkipedia (https://en.wikipedia.org/wiki/Neospora_caninum#/media/File:Neospora_caninum_ (5256961091).jpg).
